# Dietary and Circulating Advanced Glycation End Products in Relation to Inflammation and Cognitive Function in Healthy Young Adults

**DOI:** 10.3390/nu18203442

**Published:** 2026-10-20

**Authors:** Mevra Aydin Cil, Neva Karatas, Fatma Betül Ozgeris, Esen Tasgin, Serap Sari, Rüveyda Esra Özkalaycı

**Affiliations:** 1 Department of Nutrition and Dietetics, Faculty of Health Sciences, Atatürk University, 25240 Erzurum, Türkiye; mevra.aydin@atauni.edu.tr (M.A.C.); ngungor@atauni.edu.tr (N.K.); betul.ozgeris@atauni.edu.tr (F.B.O.); esent25@atauni.edu.tr (E.T.); 2 Department of Psychiatry, Faculty of Medicine, Atatürk University, 25240 Erzurum, Türkiye; serap.sari@atauni.edu.tr; 3 Department of Nutrition and Dietetics, Faculty of Health Sciences, Recep Tayyip Erdogan University, 53020 Rize, Türkiye

**Keywords:** AGEs, sRAGE, dAGEs, inflammation, cognitive performance

## Abstract

**Background:** Advanced glycation end products (AGEs) may link diet, systemic inflammation, and cognitive function; however, evidence in healthy young adults is limited. **Objective:** This study aimed to examine the associations of dietary AGE (dAGE) intake, circulating AGE–RAGE biomarkers, inflammation, and oxidant–antioxidant markers with cognitive performance. **Methods:** This cross-sectional study included healthy young adults aged 19–26 years. dAGE intake was estimated using a food-frequency questionnaire (FFQ) and a three-day food record (3D-FR). Serum AGEs (sAGEs), soluble RAGE (sRAGE), interleukin-6, tumour necrosis factor-alpha (TNF-α), malondialdehyde, catalase, superoxide dismutase, and glutathione were measured. Cognitive performance was assessed using Digit Span Forward, Backward, and Total scores and Stroop Interference. Separate multivariable linear regression models were adjusted for age, sex, body mass index, and Beck Depression Inventory-II scores, with Benjamini–Hochberg false-discovery-rate correction. Covariate-adjusted partial least squares structural equation modelling (PLS-SEM) examined the prespecified sAGE–sRAGE–inflammation pathways. **Results:** In the correlation analyses, dAGE intake estimated using the FFQ and 3D-FR was not significantly associated with sAGE concentrations (*p* > 0.05). In the separate regression models, higher sAGEs (β = −0.36, 95% CI −0.54 to −0.16) and TNF-α (β = −0.33, 95% CI −0.53 to −0.13) were associated with poorer Digit Span Backward performance after false-discovery-rate correction. No other cognitive associations remained significant. In PLS-SEM, sAGEs were positively associated with sRAGE (β = 0.505, *p* < 0.001), and sRAGE was positively associated with inflammation (β = 0.601, *p* < 0.001). Greater inflammation was associated with poorer Digit Span Backward performance (β = −0.398, 95% CI −0.697 to −0.110, *p* = 0.009), whereas the direct sRAGE–Digit Span Backward path was not significant. An indirect inverse association between sRAGE and Digit Span Backward through inflammation was supported (β = −0.239, 95% bootstrap CI −0.457 to −0.057). **Conclusions**: In conclusion, higher circulating sAGEs and TNF-α concentrations were associated with poorer working-memory performance, particularly on Digit Span Backward, in healthy young adults, whereas estimated dAGE intake showed no significant associations with cognitive performance. Longitudinal studies with larger samples are needed to establish temporality and confirm the significance of these associations.

## 1. Introduction

Cognitive health is a fundamental component of healthy aging and encompasses various domains such as attention, memory, learning, executive functions, and processing speed [1,2]. Although clinically evident cognitive decline, particularly in fluid abilities, typically becomes more prominent in later adulthood and significantly accelerates during the terminal phase of life, the trajectories of cognitive vulnerability are shaped by individual circumstances established much earlier, consistent with a life-span development perspective [3]. Low-grade systemic inflammation, oxidative stress, and metabolic dysfunction have been associated with poorer cognitive function throughout life [4,5,6,7]. Therefore, identifying modifiable factors related to these processes during young adulthood may contribute to a lifelong approach to preserving cognitive health [8]. Among these modifiable factors, nutrition has attracted considerable attention due to its potential to modulate metabolic, inflammatory, and oxidative pathways involved in the maintenance of cognitive health [9].

Advanced glycation end products (AGEs) represent one potential biochemical link between diet, systemic metabolism, and cognitive function [10]. AGEs comprise a heterogeneous group of predominantly irreversible compounds formed through non-enzymatic reactions between reducing sugars and proteins, lipids, or nucleic acids [11]. Their formation begins with a reversible Schiff base, followed by the generation of Amadori products and subsequent oxidative rearrangements leading to stable AGE compounds [12]. AGEs are produced endogenously, particularly under conditions of hyperglycaemia and oxidative stress, but they are also obtained exogenously through the diet [11,12,13]. Foods processed or cooked at high temperatures using dry-heat methods, including frying, grilling, roasting, and baking, generally contain higher AGE concentrations than foods prepared using moist-heat methods [13,14]. Dietary AGE (dAGE) intake is potentially modifiable through food selection and cooking practices, making it relevant to nutritional research. Although dAGEs undergo gastrointestinal absorption and contribute to systemic pools, the quantitative relationship between dietary intake and circulating AGE concentrations remains unclear [15].

The biological effects of AGEs are partly mediated through the receptor for advanced glycation end products (RAGE) [16]. The interaction between AGEs and membrane-bound RAGE activates intracellular signaling pathways, including nuclear factor kappa B, promotes the production of reactive oxygen species (ROS), and stimulates the expression of pro-inflammatory mediators such as interleukin-6 (IL-6) and tumour necrosis factor alpha (TNF-α) [17,18]. The AGE–RAGE axis promotes endothelial dysfunction, mitochondrial dysfunction, and chronic low-grade inflammation, establishing a self-perpetuating cycle in which endothelial injury is accompanied by excessive ROS production, further amplifying cellular damage [19]. Oxidative stress occurs when the production of ROS exceeds the capacity of antioxidant defence systems, including superoxide dismutase (SOD), catalase, and glutathione (GSH), potentially leading to oxidative damage to cellular components, particularly lipids, as reflected by increased levels of malondialdehyde (MDA), a marker of lipid peroxidation [20]. AGE–RAGE signaling may contribute to this imbalance by promoting ROS generation and inflammatory responses, while increased oxidative stress may further accelerate AGE formation [21].

Moreover, AGE–RAGE signaling impairs mitochondrial function through the upregulation of c-Jun *N*-terminal kinase (JNK), thereby sustaining inflammatory signaling and ultimately contributing to cell death [22]. In neural tissue, these processes may promote neuroinflammation, disrupt synaptic plasticity, and adversely affect neuronal function [23]. The soluble receptor for advanced glycation end products (sRAGE) encompasses circulating RAGE isoforms that retain the extracellular ligand-binding domain but lack the transmembrane and cytoplasmic domains required for intracellular signal transduction. Total circulating sRAGE comprises cleaved RAGE (cRAGE), generated through proteolytic shedding of membrane-bound RAGE, and endogenous secretory RAGE (esRAGE), produced through alternative splicing of RAGE pre-mRNA [24,25,26]. Although sRAGE has traditionally been regarded as a decoy receptor that sequesters pro-inflammatory RAGE ligands and limits their interaction with membrane-bound RAGE, evidence concerning its relationship with inflammation remains inconsistent [25,26]. Because inflammatory stimuli can promote the proteolytic shedding of membrane-bound RAGE, elevated serum sRAGE may indicate not only enhanced ligand sequestration but also sustained cell-surface RAGE activation and an ongoing state of low-grade chronic inflammation. Moreover, sRAGE may interact with the leukocyte integrin MAC-1 and thereby facilitate leukocyte recruitment and sustain inflammation in certain acute settings [25]. Conversely, the observation that healthy centenarians exhibit higher circulating sRAGE concentrations than healthy younger individuals has led to the proposition that sRAGE may also be associated with healthy aging and exceptional longevity [25,27]. In this study, total sRAGE was included as a complementary biomarker of the AGE-RAGE axis because its circulating concentration may reflect both ligand sequestration and membrane-RAGE shedding [24,25,28]. Therefore, its assessment together with serum AGEs and inflammatory cytokines provides information about receptor-related processes beyond AGEs burden alone.

Young adulthood represents an important period for the establishment of dietary behaviours and long-term cognitive health [29,30]. University students may be particularly susceptible to irregular meal patterns, frequent consumption of processed or fast foods, examination-related stress, and disrupted sleep, all of which may influence dAGE intake and cognitive performance [31,32,33]. Previous studies have examined dietary AGE intake, circulating AGE–RAGE-related markers in relation to cognitive performance, cognitive decline, or cognitive impairment in older adults, individuals with type 2 diabetes, and patients with cognitive disorder [34,35,36,37,38]. Specifically, Beeri et al. [39] reported that higher dietary AGE intake was associated with faster global cognitive decline, particularly in episodic memory and perceptual speed, among 684 older adults; however, circulating AGE–RAGE biomarkers were not assessed. West et al. [40] investigated both dietary AGE intake and serum methylglyoxal derivatives in 49 older adults, reporting associations with faster decline in memory and attention, respectively, but did not assess sRAGE or oxidative–antioxidative biomarkers. Taken together, these findings indicate that the relationships of dietary AGE intake and circulating AGE–RAGE biomarkers with cognitive performance remain insufficiently characterized in healthy young adults, particularly in relation to systemic inflammation and oxidative–antioxidative status. The present study addresses this gap by examining the associations of dietary AGE intake and circulating AGE–RAGE biomarkers with working memory and executive function, and by investigating their relationships with inflammatory and oxidative–antioxidative biomarkers in healthy young adults. We hypothesized that dietary AGE intake, circulating AGE–RAGE biomarkers, systemic inflammation, and oxidative–antioxidative status would be associated with cognitive performance.

## 2. Materials and Methods

### 2.1. Study Design and Participants

This cross-sectional study included healthy young adults enrolled at the Faculty of Health Sciences, Atatürk University, Erzurum, Türkiye. Participant recruitment and data collection were conducted between November 2023 and April 2024.

**Figure 1:**
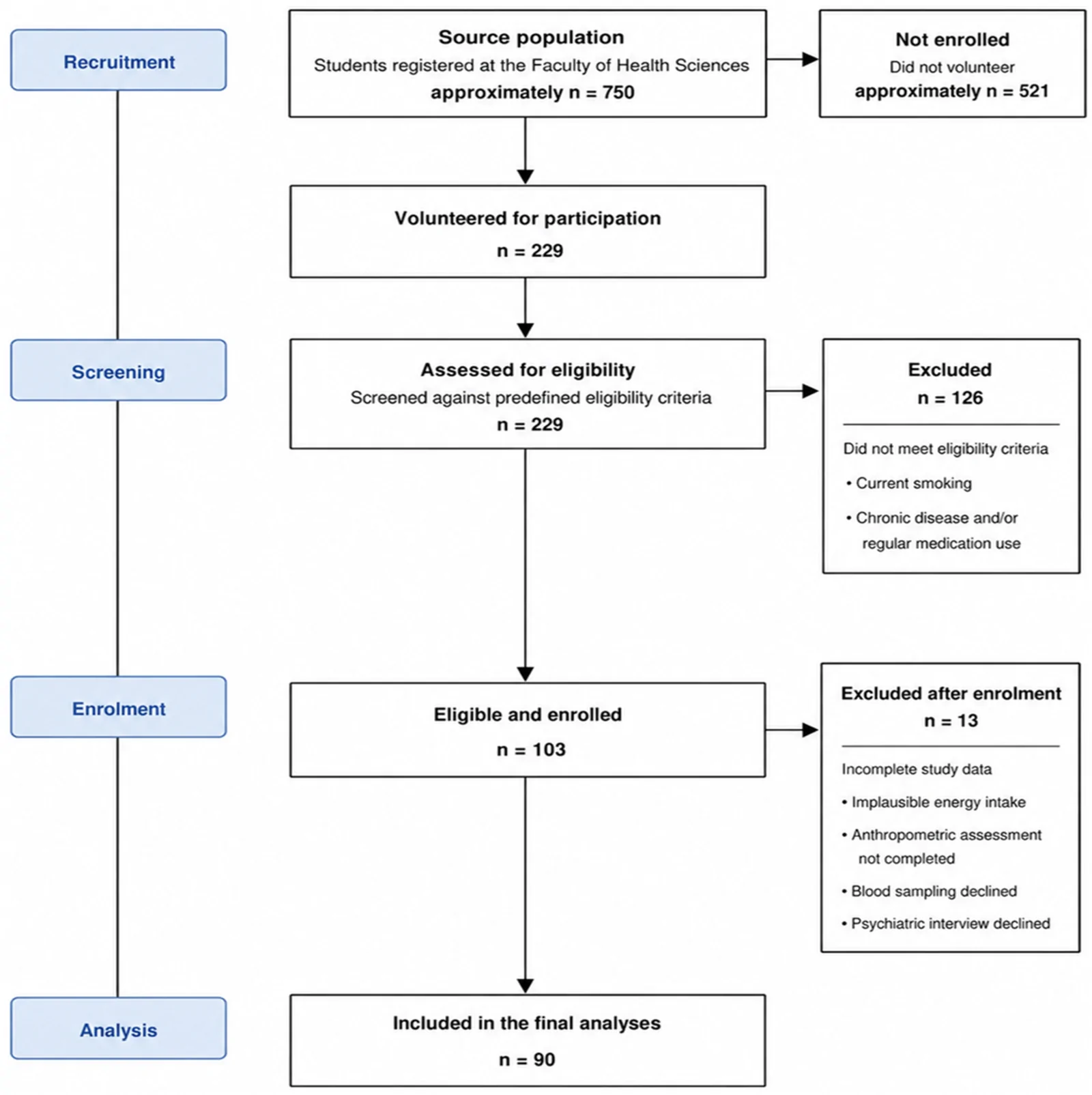
Flowchart of participant selection process.

An a priori power analysis was conducted using G*Power (version 3.1.9.7) based on a two-tailed correlation analysis. Assuming a moderate correlation of r = 0.30, an alpha level of 0.05, and 80% statistical power, the minimum required sample size was 84 participants. Approximately 750 students were registered at the Faculty during the recruitment period, of whom 229 volunteered and were screened for eligibility. Students were eligible if they were aged 19–26 years, were currently enrolled at the Faculty, and agreed to complete the dietary, anthropometric, biochemical, cognitive, and depressive symptom assessments. The exclusion criteria were current smoking, the presence of a self-reported chronic or acute disease, and regular medication use. Following eligibility screening, 103 participants were enrolled. Participants were subsequently excluded if they had incomplete or implausible dietary intake records (<500 or >3500 kcal/day for women and <800 or >4000 kcal/day for men), did not complete the anthropometric assessment, or declined blood sampling or the psychiatric interview. The final complete-case analytical sample comprised 90 participants. For the PLS-SEM analysis, sample-size adequacy was additionally evaluated using the inverse square root method proposed by Kock and Hadaya [41]. With a final sample of 90 participants, a significance level of 0.05, and 80% statistical power, the post hoc assessment indicated sensitivity to structural path coefficients of approximately |β| ≥ 0.26. The participant-selection process is presented in Figure 1.

The study was conducted in accordance with the latest adaptation of the Helsinki Declaration and was approved by the Ethics Committee of the Atatürk University Health Science Faculty (No: 2022-04-19; Date: 27 April 2022). All participants received detailed information about the study procedures and provided written informed consent before participation.

### 2.2. Study Procedures

All assessments were conducted by trained researchers following standardized procedures. Participants completed socio-demographic, dietary, depressive symptom, and cognitive assessments. Anthropometric measurements were obtained, and venous blood samples were collected for the determination of sAGEs, sRAGE, inflammatory biomarkers, and oxidative–antioxidative markers.

#### 2.2.1. Anthropometric Measurements

Body weight was measured with participants wearing light clothing and no footwear, using a calibrated electronic scale to the nearest 0.1 kg (Seca, Hamburg, Germany). Height was measured without footwear using a wall-mounted stadiometer to the nearest 0.1 cm (Seca, Hamburg, Germany). Body mass index (BMI) was calculated as body weight in kilograms divided by height in metres squared (kg/m^2^) [42].

#### 2.2.2. Dietary Assessment and Estimation of dAGE Intake

Dietary intake was assessed using both a food-frequency questionnaire (FFQ) and a three-day food record (3D-FR). The validated 229-item FFQ was administered by a trained dietitian to estimate food consumption during the previous year [43]. For each food and beverage item, consumption frequency, usual portion size (g or mL), and typical cooking methods (e.g., boiling, grilling, frying, and baking) were recorded. Portion sizes were estimated using a photographic food atlas [44]. For the 3D-FR, participants prospectively recorded all foods and beverages consumed on two weekdays and one weekend day. Participants received standardized verbal and written instructions regarding the recording of food type, portion size, preparation method, brand, and cooking technique. Dietary records were reviewed by a trained dietitian together with each participant to clarify incomplete or ambiguous entries. Daily energy and nutrient intakes were calculated using the Nutrition Information System (BeBiS, version 8.0).

Dietary AGE intake was estimated separately from the FFQ and 3D-FR using the food AGE database developed by Uribarri et al. [45]. In the absence of a dietary AGE database specifically developed for the Turkish population, this database was used as the reference for dAGE estimation. Each reported food was matched to the database according to food type and, where available, cooking and processing methods; when an exact match was unavailable, the most comparable database item was selected based on its composition and preparation method. For mixed dishes, foods were disaggregated into their individual ingredients according to the reported recipe, and dAGEs values were assigned to each component separately. When more than one dAGEs value was available for the same food item, the mean of the available values was used. Daily dAGE intake was expressed as kilounits per day (kU/day). The FFQ-derived and 3D-FR-derived dAGE estimates were analysed separately because they represented habitual and short-term dietary intake, respectively.

#### 2.2.3. Blood Collection and Biochemical Analyses

Venous blood samples were collected in the morning following an overnight fast of 8–12 h. Samples were centrifuged at 4000 rpm for 10 min at 4 °C, and the serum was stored at −20 °C until analysis. sAGE (cat No. 201-12-0004), sRAGE (cat No. 201-12-0032), TNF-α (cat No. 201-12-0083), and IL-6 (cat No. 201-12-0091) concentrations were measured using commercially available human enzyme-linked immunosorbent assay (ELISA) kits (SunRed Biotechnology Company, Shanghai, China) according to the manufacturer’s instructions. Serum samples were diluted 1:20 prior to analysis, and the concentrations obtained from the standard curves were corrected according to the dilution factor. Concentrations were expressed as ng/mL for serum AGEs, pg/mL for sRAGE, and ng/L for TNF-α and IL-6. The measurement ranges and analytical sensitivities were 2–20 ng/mL and 1.125 ng/mL for sAGE, 20–6000 pg/mL and 18.725 pg/mL for sRAGE, 3–900 ng/L and 2.827 ng/L for TNF-α, and 3–600 ng/L and 2.112 ng/L for IL-6, respectively. The intra-assay and inter-assay coefficients of variation (CVs) were <10% and <12%, respectively, for all four ELISAs.

Serum SOD and catalase activities and GSH and MDA levels were determined spectrophotometrically. Serum SOD and catalase activities were determined using the methods described by Sun et al. [46] and Góth [47], respectively. Serum GSH levels were measured using the DTNB method [48], and MDA levels were determined using the thiobarbituric acid-reactive substances method [49].

#### 2.2.4. Cognitive Assessment

Cognitive performance was assessed using the Digit Span subtest of the Wechsler Adult Intelligence Scale–Revised (WAIS-R) [50] and the Stroop Test TBAG Form, a Turkish adaptation of the Stroop color–word paradigm [51,52]. All tests were administered individually in a quiet setting by a trained researcher (psychiatrist) using standardized instructions.

The Turkish standardization study of the WAIS-R was conducted by Sezgin et al. [53]. The Digit Span Test consists of forward and backward components: The former requires subjects to repeat verbally presented number sequences in their original order, while the latter requires recalling the number sequence in reverse order.

Sequence length increased progressively, and administration was discontinued according to the standard stopping rule. The Digit Span Forward Test begins with two three-digit sequences, increasing by one digit at each step until the final sequence consists of two nine-digit sequences. The Digit Span Backward Test begins with two two-digit sequences, increasing by one digit at each step until the final sequence consists of two eight-digit sequences. One point is awarded for each correctly repeated sequence, and 0 points for each incorrectly repeated sequence. The total score a participant can receive on the Digit Span Forward Test is 14 points, the same as on the Digit Span Backward Test. The maximum total score on the Digit Span Test is 28.

The Digit Span Forward Test score primarily reflected immediate auditory-verbal attention and short-term storage, whereas the Digit Span Backward Test additionally required maintaining and mentally manipulating information and was therefore interpreted as a measure of verbal working-memory performance. The Digit Span Test Total Score was calculated by summing the scores of both subtests, with higher scores indicating better cognitive performance [53,54].

Inhibitory control and resistance to cognitive interference were assessed using the Stroop Test TBAG Form, a Turkish adaptation and standardisation of the Stroop color–word paradigm [51]. The Stroop Test TBAG Form consists of five tasks. The test assesses selective attention and response inhibition through tasks such as reading words on cards and naming the ink colors used to print the circles or words. In all five tasks, completion time, number of errors, and number of corrections are recorded according to standardized instructions. In the fifth test task, the participant is asked to name the ink colors used to print the color names on the card. By requiring the suppression of the dominant automated word-reading response, the fifth task assesses response inhibition, an executive function related to the frontal lobe that reflects the ability to resist automated processes. Performance on the fifth task was operationalized as completion time, with longer durations indicating greater interference and reduced inhibitory capacity [51].

#### 2.2.5. Depressive Symptoms

Depressive symptoms were assessed using the Turkish version of the Beck Depression Inventory-II (BDI-II), originally developed by Beck et al. [55] and validated in Turkish adults by Kapci et al. [56]. The BDI-II is a 21-item self-report instrument assessing depressive symptoms over the preceding two weeks. Each item is scored from 0 to 3, yielding a total score ranging from 0 to 63, with higher scores indicating greater depressive-symptom severity. The Turkish version demonstrated high internal consistency in a non-clinical adult sample (Cronbach’s α = 0.90) [56]. The total BDI-II score was included as a continuous covariate in the adjusted analyses.

### 2.3. Statistical Analysis

Statistical analyses were performed using IBM SPSS Statistics version 27.0 (IBM Corp., Armonk, NY, USA), R version 4.6.4 (R Foundation for Statistical Computing, Vienna, Austria), and SmartPLS version 4.0.8.3 (SmartPLS GmbH, Oststeinbek, Germany). Distributions of continuous variables were examined using the Shapiro–Wilk test, histograms, and Q–Q plots. Skewness and kurtosis values between −2 and +2 were considered to indicate normal distribution [57]. Normally distributed continuous variables are presented as mean ± standard deviation, non-normally distributed variables as median and interquartile range, and categorical variables as frequencies and percentages. Pairwise correlations were assessed using Pearson’s correlation coefficient for normally distributed variables and Spearman’s rank correlation coefficient for pairs involving TNF-α or IL-6, which were not normally distributed. To account for multiple comparisons, the Benjamini–Hochberg false discovery rate (FDR) correction was applied across all 120 pairwise correlation tests. Correlations with FDR-adjusted *p*-values (q < 0.05) were considered statistically significant.

Separate multivariable linear regression models were constructed for each exposure–cognitive outcome combination. FFQ-derived dAGEs, 3D-FR-derived dAGEs, serum AGEs, sRAGE, TNF-α, IL-6, MDA, catalase, SOD, and GSH were entered individually and were not mutually adjusted. All models were adjusted a priori for age, sex, BMI, and BDI-II score. Model assumptions were evaluated by examining residual normality, linearity, homoscedasticity, influential observations, and multicollinearity. Results are presented as standardized regression coefficients (β), 95% confidence intervals (CIs), and *p*-values. To account for multiple testing, the Benjamini–Hochberg procedure was applied across all 40 exposure–outcome comparisons. Statistical significance was defined as an FDR-adjusted *p*-value < 0.05.

PLS-SEM was selected as a complementary exploratory approach to simultaneously examine the proposed interrelationships among dAGE intake, circulating AGEs, sRAGE, inflammation, and cognitive performance. Compared with covariance-based SEM (CB-SEM), PLS-SEM is particularly suitable when the research objective is prediction-oriented or exploratory and when the structural model involves multiple interrelated pathways. In addition, PLS-SEM does not require multivariate normality, making it appropriate for data that may deviate from normality [58]. FFQ and 3D-FR dAGE estimates were represented as separate single-indicator constructs, while sAGEs and sRAGE were specified as single-indicator constructs. Systemic inflammation was specified as a formative construct comprising IL-6 and TNF-α. Indicator collinearity was assessed using variance inflation factors. The relevance of the formative indicators was evaluated using outer weights, bootstrap significance, and absolute outer loadings.

The structural model specified paths from FFQ-derived and 3D-FR-derived dAGEs to sAGEs, from sAGEs to sRAGE, from sRAGE to inflammation, and from inflammation to cognitive performance. A direct path from sRAGE to cognitive performance was also estimated. Age, sex, BMI, and BDI-II score were included a priori as covariates with direct paths to inflammation and the corresponding cognitive outcome.

Separate PLS-SEM models were estimated for Digit Span Backward, Digit Span Total, Digit Span Forward, and Stroop Interference. Structural-model results are reported as standardized path coefficients (β), bootstrap standard errors, t-statistics, 95% percentile bootstrap CIs, *p*-values, coefficients of determination (R^2^), and local effect sizes (f^2^). Statistical inference was based on non-parametric bootstrapping with 10,000 resamples. Specific indirect effects were also estimated using bootstrapping.

Out-of-sample predictive performance was evaluated using PLSpredict with 10-fold cross-validation and 10 repetitions. Predictive performance was assessed using Q^2^predict, root mean square error, and mean absolute error, with predictions from a linear model used as the benchmark. The cross-validated predictive ability test was additionally used to compare prediction loss between the PLS-SEM and linear-model benchmarks. The standardized root mean square residual was reported as a supplementary model-fit index. Analyses were based on complete cases, and all statistical tests were two-sided.

## 3. Results

### 3.1. General Characteristics of Participants

A total of 90 healthy young adults were included in the study, of whom 26.7% were male and 73.3% were female. The mean age was 22.2 ± 1.3 years, and the mean BMI was 22.2 ± 3.2 kg/m^2^. Mean dAGE intake was determined to be 14,067.7 ± 6516.8 kU/day and 10,168.5 ± 5409.7 kU/day as measured by the FFQ and the 3-day food record, respectively. The mean serum AGE and sRAGE levels were 308.9 ± 189.6 ng/mL and 1140.9 ± 787.9 pg/mL, respectively, while the median TNF-α and IL-6 concentrations were 157.1 [123.5] ng/L and 16.0 [8.2] ng/L, respectively. The mean Digit Span Total score was 13.8 ± 3.6, and the mean Stroop Interference score was 18.1 ± 3.9. Detailed participant characteristics are presented in Table 1.

### 3.2. Correlation Analyses

**Figure 2:**
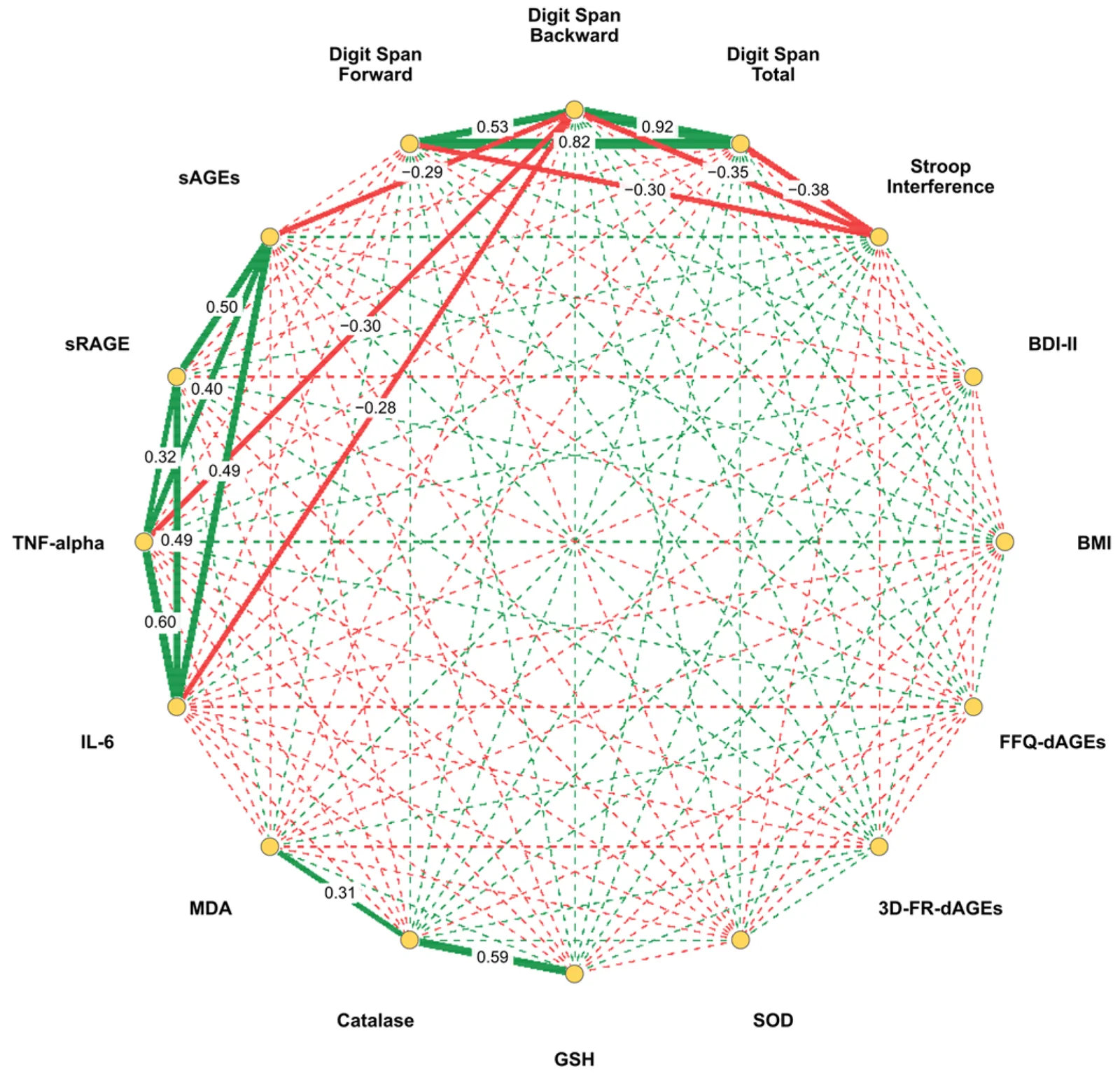
Correlation network among cognitive performance, dAGEs, sAGEs, sRAGE, inflammatory markers, and oxidative stress biomarkers. Nodes represent study variables, and edges represent pairwise correlations. Spearman’s rank correlation coefficients (ρ) were used for all correlations involving TNF-α or IL-6, whereas Pearson correlation coefficients (r) were used for all other variable pairs. Green edges indicate positive correlations, whereas red edges indicate negative correlations. Solid lines represent statistically significant correlations after Benjamini–Hochberg false discovery rate (FDR) correction (q < 0.05), whereas dashed lines represent non-significant correlations (q ≥ 0.05). Edge thickness is proportional to the absolute correlation coefficient (|r| or |ρ|). Correlation coefficients are displayed only for associations that remained statistically significant after FDR correction. BDI-II, Beck Depression Inventory-II; BMI, body mass index; FFQ, food frequency questionnaire; GSH, glutathione; IL-6, interleukin-6; MDA, malondialdehyde; sAGEs, serum advanced glycation end products; sRAGE, soluble receptor for advanced glycation end products; SOD, superoxide dismutase; TNF-α, tumour necrosis factor-alpha; 3D-FR-dAGEs, dietary advanced glycation end products estimated from 3-day food records; FFQ-dAGEs, dietary advanced glycation end products estimated from the food frequency questionnaire.

As shown in Figure 2, correlation analyses revealed significant positive associations among AGE–RAGE and inflammatory biomarkers. sAGE concentrations were positively correlated with sRAGE (r = 0.505, *p* < 0.001), TNF-α (ρ = 0.397, *p* < 0.001), and IL-6 (ρ = 0.488, *p* < 0.001). Similarly, sRAGE was positively correlated with TNF-α (ρ = 0.318, *p* = 0.002) and IL-6 (ρ = 0.487, *p* < 0.001), while TNF-α and IL-6 were positively correlated with each other (ρ = 0.602, *p* < 0.001). Regarding cognitive performance, higher sAGE concentrations were negatively correlated with Digit Span Backward scores (r = −0.291, *p* = 0.005). Higher TNF-α and IL-6 concentrations were also negatively correlated with Digit Span Backward scores (ρ = −0.301, *p* = 0.004 and ρ = −0.283, *p* = 0.007, respectively). All reported correlations remained statistically significant after Benjamini–Hochberg false discovery rate (FDR) correction (q < 0.05). No significant correlations were observed between dietary AGE intake, assessed using the FFQ or 3-day food records, and circulating AGE–RAGE biomarkers or cognitive outcomes after FDR correction.

### 3.3. Regression Analyses

**Figure 3:**
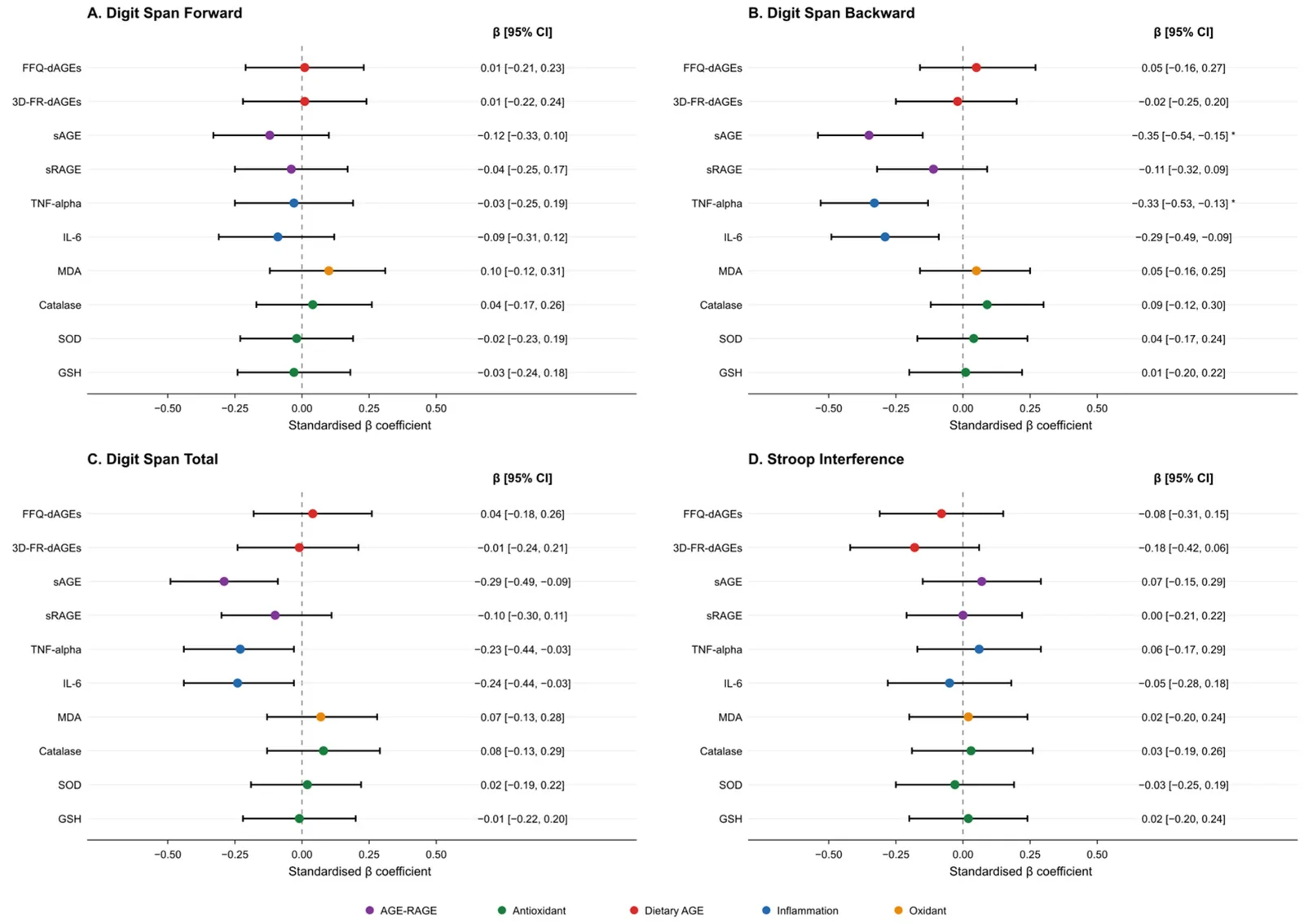
Associations of dietary advanced glycation end products (dAGEs), circulating AGE–RAGE biomarkers, inflammatory biomarkers, oxidative stress markers, and antioxidant defence markers with cognitive performance. Separate multivariable linear regression models were performed for (**A**) Digit Span Forward, (**B**) Digit Span Backward, (**C**) Digit Span Total, and (**D**) Stroop Interference. Each model included one exposure variable and was adjusted for age, sex, body mass index, and Beck Depression Inventory-II score. Points represent standardized regression coefficients (β), and horizontal lines indicate 95% confidence intervals (95% CIs). Red symbols indicate dietary AGEs (FFQ-dAGEs and 3D-FR-dAGEs), purple symbols indicate circulating AGE–RAGE biomarkers (sAGEs and sRAGE), blue symbols indicate inflammatory biomarkers (TNF-α and IL-6), orange symbols indicate the oxidative stress marker (MDA), and green symbols indicate antioxidant defence markers (catalase, SOD, and GSH). BDI-II, Beck Depression Inventory-II; GSH, glutathione; IL-6, interleukin-6; MDA, malondialdehyde; sAGEs, serum advanced glycation end products; sRAGE, soluble receptor for advanced glycation end products; SOD, superoxide dismutase; TNF-α, tumour necrosis factor-alpha; 3D-FR-dAGEs, dietary advanced glycation end products estimated from 3-day food records; FFQ-dAGEs, dietary advanced glycation end products estimated from the food frequency questionnaire. Displayed *p*-values were adjusted for multiple comparisons using the Benjamini–Hochberg false discovery rate procedure. * FDR-adjusted *p* < 0.05.

Figure 3 summarizes the associations between dietary AGEs, circulating AGE–RAGE biomarkers, inflammatory biomarkers, oxidative stress markers, and antioxidant defence markers with cognitive performance based on separate multivariable linear regression models adjusted for age, sex, BMI, and Beck Depression Inventory score. After Benjamini–Hochberg FDR correction, higher sAGEs (β = −0.35, 95% CI [−0.54, −0.15], FDR-adjusted *p* < 0.05) and TNF-α concentrations (β = −0.33, 95% CI [−0.53, −0.13], FDR-adjusted *p* < 0.05) were significantly associated with lower Digit Span Backward scores in separate models adjusted for age, sex, BMI, and BDI-II score. No other FDR-significant associations were observed across the cognitive outcomes examined (Figure 3).

### 3.4. PLS-SEM

Assessment of the formative measurement model indicated no problematic collinearity between the inflammatory indicators (VIF = 1.781 for both IL-6 and TNF-α). IL-6 contributed significantly to the inflammation construct (outer weight = 0.808, *p* < 0.001; outer loading = 0.981). Although the outer weight of TNF-α was not statistically significant (outer weight = 0.260, *p* = 0.350), its absolute outer loading was satisfactory (0.796). TNF-α was therefore retained because of its theoretical and content relevance to systemic inflammation and its substantial absolute loading, despite IL-6 contributing more strongly to the formative construct (Supplementary Table S1).

**Figure 4:**
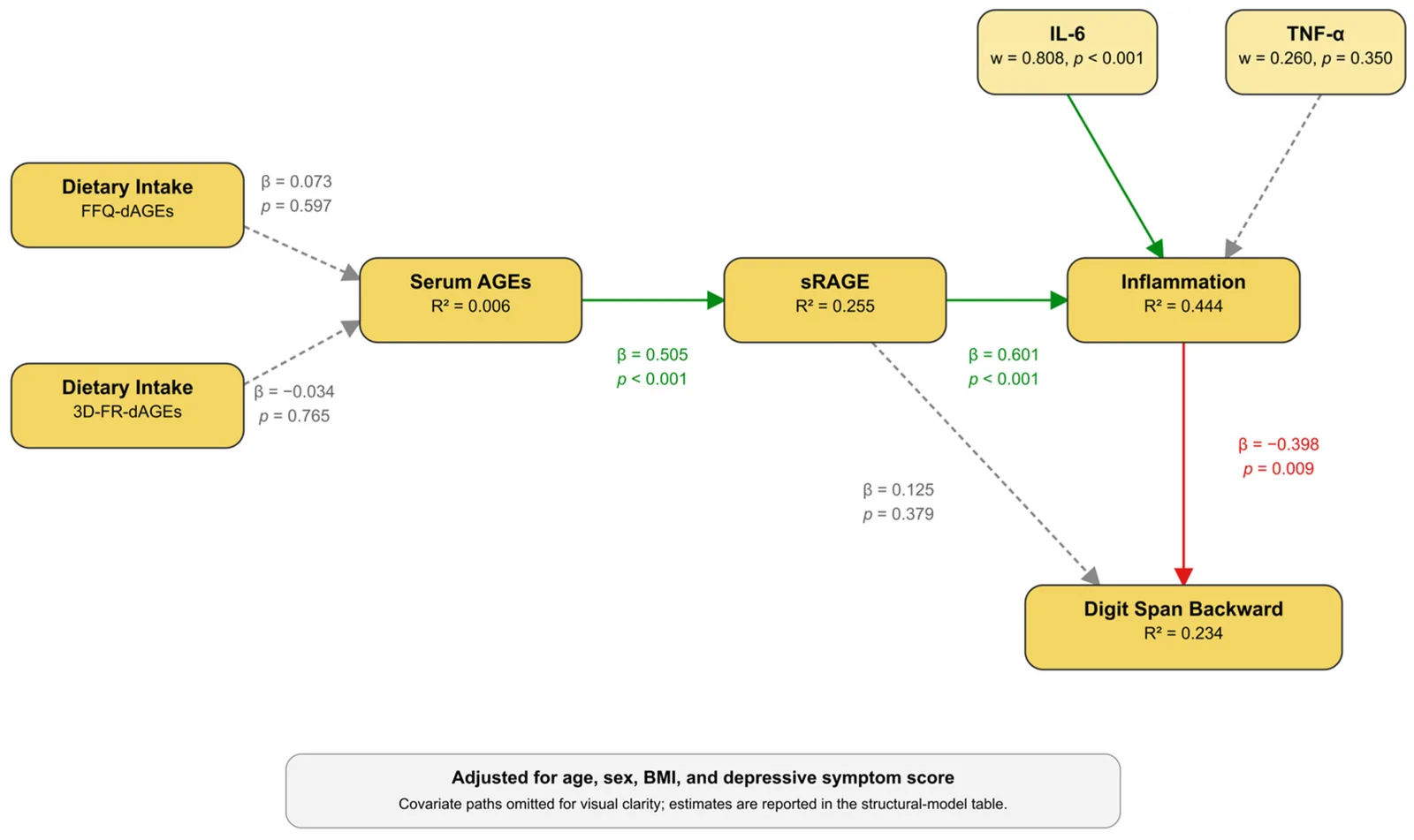
Adjusted partial least squares structural equation model linking dietary advanced glycation end-product intake, circulating AGEs, inflammation, and working-memory performance. Values adjacent to the structural paths represent standardized path coefficients (β), whereas values within endogenous constructs represent the explained variance (R^2^). Outer weights (w) are presented for the formative inflammation composite, which comprised IL-6 and TNF-α. Solid green arrows indicate statistically significant positive paths, the solid red arrow indicates a statistically significant negative path, and grey dashed arrows indicate non-significant paths. The model was adjusted for age, sex, body mass index, and BDI-II score. Covariate paths were omitted from the figure for visual clarity. BMI, body mass index; IL-6, interleukin-6; sAGEs, serum advanced glycation end products; sRAGE, soluble receptor for advanced glycation end products; TNF-α, tumour necrosis factor-alpha; 3D-FR-dAGEs, dietary advanced glycation end products estimated from 3-day food records; FFQ-dAGEs, dietary advanced glycation end products estimated from the food frequency questionnaire.

As shown in Figure 4, sAGEs were positively associated with sRAGE (β = 0.505, 95% CI 0.263–0.674; *p* < 0.001), and sRAGE was positively associated with the inflammation construct (β = 0.601, 95% CI 0.383–0.758; *p* < 0.001). Greater inflammation was associated with poorer Digit Span Backward performance (β = −0.398, 95% CI −0.697 to −0.110; *p* = 0.009). The adjusted model explained 0.6% of the variance in sAGEs, 25.5% of the variance in sRAGE, 44.4% of the variance in inflammation, and 23.4% of the variance in Digit Span Backward performance.

Table 2 presents the covariate-adjusted structural model estimates for the hypothesised pathways predicting Digit Span Backward performance. dAGE intake estimated using the FFQ and three-day food records showed no significant associations with sAGEs (β = 0.073, *p* = 0.597 and β = −0.034, *p* = 0.765, respectively). The direct path from sRAGE to Digit Span Backward was also not statistically significant (β = 0.125, 95% CI −0.154 to 0.404; *p* = 0.379).

**Table 2:** Structural model estimates for the hypothesised pathways predicting Digit Span Backward performance.

Path	β	SE	t	*p*	95% CI	f^2^
FFQ-dAGEs → serum AGEs	0.073	0.138	0.528	0.597	−0.208 to 0.332	0.005
3D-FR-dAGEs → serum AGEs	−0.034	0.113	0.299	0.765	−0.250 to 0.193	0.001
Serum AGEs → sRAGE	0.505	0.106	4.754	<0.001	0.263 to 0.674	0.342
sRAGE → inflammation	0.601	0.099	6.070	<0.001	0.383 to 0.758	0.638
Inflammation → Digit Span Backward	−0.398	0.152	2.617	0.009	−0.697 to −0.110	0.115
sRAGE → Digit Span Backward	0.125	0.142	0.881	0.379	−0.154 to 0.404	0.012

β denotes the standardized path coefficient; SE, bootstrapped standard error; t, bootstrap t statistic; CI, percentile bootstrap confidence interval; and f^2^, Cohen’s local effect size. All estimates were obtained from models adjusted a priori for age, sex, body mass index, and depressive symptom score. Statistical inference was based on two-sided tests and 95% bootstrap CIs. Values of f^2^ around 0.02, 0.15, and 0.35 were interpreted as small, medium, and large local effects, respectively. sRAGE, soluble receptor for advanced glycation end products; 3D-FR-dAGEs, dietary advanced glycation end products estimated from 3-day food records; FFQ-dAGEs, dietary advanced glycation end products estimated from the food frequency questionnaire.

As shown in Table 3, the specific indirect-effect analysis supported a positive indirect association between serum AGEs and inflammation through sRAGE (β = 0.303, 95% CI 0.107–0.487). A negative indirect association between sRAGE and Digit Span Backward through inflammation was also observed (β = −0.239, 95% CI −0.457 to −0.057). The indirect pathway from serum AGEs to Digit Span Backward through sRAGE alone was not supported (β = 0.063, 95% CI −0.082 to 0.217).

**Table 3:** Bootstrapped specific indirect effects of the PLS-SEM model predicting Digit Span Backward performance.

Specific Indirect Effect	β	SE	95% CI
sAGEs → sRAGE → Inflammation	0.303	0.099	0.107 to 0.487
sRAGE → Inflammation → Digit Span Backward	−0.239	0.102	−0.457 to −0.057
sAGEs → sRAGE → Digit Span Backward	0.063	0.074	−0.082 to 0.217
sAGEs → sRAGE → inflammation → Digit Span Backward	−0.121	0.064	−0.271 to −0.022

β denotes the standardized specific indirect effect; SE, bootstrapped standard error; and CI, percentile bootstrap confidence interval. Statistical inference was based on two-sided tests and 95% bootstrap CIs. Indirect effects were considered statistically supported when the corresponding 95% bootstrap CI excluded zero. All structural estimates were adjusted a priori for age, sex, body mass index, and depressive symptom score. Because the study was cross-sectional, the indirect effects represent statistical decomposition of associations and should not be interpreted as evidence of temporal or causal mediation. sAGEs, serum advanced glycation end products; sRAGE, soluble receptor for advanced glycation end products.

In the covariate-adjusted PLS-SEM analyses, inflammation was inversely associated with Digit Span Backward (β = −0.398, 95% CI −0.697 to −0.110; *p* = 0.009) and Digit Span Total performance (β = −0.308, 95% CI −0.596 to −0.025; *p* = 0.034). No significant associations were observed for Digit Span Forward (β = −0.099, 95% CI −0.404 to 0.235; *p* = 0.539) or Stroop interference (β = −0.051, 95% CI −0.381 to 0.284; *p* = 0.762). Complete measurement-model, covariate-adjusted structural-model, and predictive-assessment results for all cognitive outcomes are presented in the Supplementary Materials.

## 4. Discussion

This cross-sectional study examined, to our knowledge, for the first time in a healthy young-adult sample, how dAGE intake, circulating AGEs–RAGE levels, systemic inflammation, and oxidative–antioxidative status relate to attention and working-memory performance. First, dAGE intake estimated using the FFQ or the three-day food record was not associated with sAGE concentrations. Second, higher sAGEs were associated with higher sRAGE, while higher sRAGE was strongly associated with the inflammation construct comprising IL-6 and TNF-α. Third, in the separate covariate-adjusted regression models, higher sAGE and TNF-α concentrations were associated with poorer Digit Span Backward performance after FDR correction, whereas no other associations remained statistically significant. Fourth, in the covariate-adjusted PLS-SEM model, greater inflammation was associated with poorer Digit Span Backward, and an indirect inverse pathway linked sRAGE to Digit Span Backward through inflammation, although the direct sRAGE–cognition path was not significant. These findings provide preliminary evidence that selected circulating AGE-related and inflammatory biomarkers are associated with working-memory performance in healthy young adults, particularly Digit Span Backward, while no significant associations were observed for Digit Span Forward or Stroop Interference.

### 4.1. dAGE Intake and sAGE/RAGE Concentrations

Mean dAGE intake in the present sample was 14,067.7 kU/day based on the FFQ and 10,168.5 kU/day based on the three-day food record, corresponding to an approximately 28% lower estimate with the latter method. This discrepancy suggests that the two dietary assessment methods may not provide interchangeable estimates of dAGEs exposure and should therefore be interpreted separately. The three-day food record value is closely in line with the 9356 kU/day recently reported by Çil et al. [59] in a comparable healthy Turkish sample using the same short-term recording approach, and with the 8900–10,543 kU/day range reported among Turkish university students [33,60] and the 9401–9686 kU/day range reported in Iranian adults using semi-quantitative FFQs [61,62]. However, these values were lower than the mean intake of 14,700 kU/day reported among healthy adults in New York City using the 3-day food record [63]. Moreover, Vasilj et al. [64] reported mean intakes of 20,390 kU/day based on an FFQ alone and 16,707 kU/day after incorporating a home-cooking frequency questionnaire in a European sample. These differences are plausible, as diets rich in processed meats and foods prepared using dry-heat methods, such as frying, grilling, and roasting, generally contain higher AGE levels than diets relying more heavily on moist-heat cooking methods. Differences in food choices and cooking practices across populations may therefore partly explain the variation in dAGE intake reported across studies [14]. Nevertheless, cross-population comparisons should be interpreted cautiously, as dAGE estimates are influenced by the dietary assessment tool, the scope of the underlying AGE database, and the degree to which cooking methods are accounted for. The internal discrepancy between our own FFQ- and 3D-FR-derived estimates is itself informative on this point. The higher dAGE estimates obtained from the FFQ than from the 3D-FR may partly reflect methodological differences between dietary assessment approaches, as considerable variation in estimated dAGE intake according to the dietary assessment method has been reported [65].

Previous studies have reported inconsistent findings regarding the relationship between dAGEs and sAGE/RAGE. In the present study, dAGE intake estimated independently using an FFQ and a 3D-FR was not significantly associated with sAGE/RAGE. While the consistency of findings across two different dietary assessment methods supports the robustness of this observation, both methods are subject to measurement error and provide estimates, not direct measurements, of actual AGE exposure. Therefore, methodological limitations in dietary AGE assessment cannot be ruled out as a possible explanation for the lack of association. This finding is consistent with that of Vasilj et al. [64], who reported no significant association between serum CML concentrations and dAGE intake estimated using an FFQ or FFQ combined with a home-cooking frequency questionnaire. However, dAGE intake was significantly associated with sRAGE, and this association was observed exclusively when dAGE intake was estimated using a home-cooking frequency questionnaire [64]. Similarly, Alipour et al. [66] reported no significant association between FFQ-derived dAGE intake and sAGEs measured using ELISA in premenopausal Iranian women with and without breast cancer. Semba et al. [67] found that although differences in dietary AGE content affected serum and urinary CML concentrations, they did not result in significant changes in circulating sRAGE or esRAGE concentrations. These findings suggest that the relationship between dAGE intake and sRAGE may depend partly on the dietary assessment method and the consideration of cooking practices. Moreover, circulating sRAGE is influenced by RAGE expression and proteolytic shedding and therefore may not directly reflect dAGE intake. Conversely, other observational and intervention studies have found that higher dAGE intake is associated with increased circulating or urinary concentrations of specific AGE compounds. For example, Linkens et al. [68] demonstrated that a high-AGE diet increased free CML, CEL, and MG-H1 concentrations in plasma and urine, although protein-bound plasma AGE concentrations remained unchanged. In another study, Kim et al. [69] reported that the effects of dietary patterns differed according to the circulating AGE compound measured, with a high-AGE dietary pattern increasing plasma CEL but not MG-H1 concentrations. Although a proportion of dAGEs is absorbed from the gastrointestinal tract, the systemic availability of individual AGE compounds varies considerably according to their chemical structure, food matrix, digestibility, gut microbial metabolism, renal clearance, subsequent tissue uptake, and the metabolic characteristics of the study population. Furthermore, circulating AGE concentrations are influenced not only by dietary intake but also by endogenous glycation and oxidative processes [11,65,70,71,72]. Differences across studies may reflect not only the dietary assessment method and the consideration of cooking practices but also the specific AGE biomarker measured and whether its free or protein-bound form was assessed.

In the present study, sAGEs were positively associated with serum sRAGE concentrations. A similar positive and independent association has been previously demonstrated in a general population of 184 nondiabetic individuals [73]. This positive relationship has also been reported among adults with obesity and patients with type 2 diabetes [74,75], suggesting that circulating sRAGE increases in response to a greater AGE burden. Increased AGE exposure may enhance the expression and turnover of membrane-bound RAGE, promoting its proteolytic shedding and thereby elevating circulating sRAGE concentrations. Consequently, this increase may reflect a cellular response to a greater AGEs burden rather than an exclusively protective mechanism. However, because total sRAGE was measured in the present study rather than its individual isoforms, the relative contributions of receptor shedding and alternative splicing (which generates esRAGE) cannot be distinguished [24,25,28,73]. In contrast, Phimphilai et al. [76] and Gateva et al. [77] found no significant association between serum pentosidine, a specific AGE compound, and sRAGE concentrations. These inconsistent findings may partly reflect differences in the specific AGE compounds measured, analytical methods, and study populations; for instance, Aroni et al. [78] reported an inverse association between CML and sRAGE, in patients with end-stage renal disease undergoing hemodialysis further indicating that the direction of this relationship varies according to these methodological and metabolic factors.

### 4.2. dAGE Intake, sAGE/RAGE, Inflammation and Oxidative/Antioxidative Parameters

In this study, serum AGE and sRAGE concentrations were found to be positively correlated with inflammatory biomarkers such as IL-6 and TNF-α. Although soluble RAGE forms are generally considered to limit membrane-bound RAGE signaling by sequestering circulating ligands, their role in inflammation is complex [17]. Interaction of sAGEs with membrane-bound RAGE can activate NF-κB, increase reactive oxygen species production, and stimulate the expression of pro-inflammatory cytokines, including IL-6 and TNF-α [79]. The increase in sAGEs burden may exceed the ligand-binding and sequestration capacity of soluble RAGE isoforms. The positive correlations between sRAGE and inflammatory biomarkers observed in the present study may reflect a compensatory response to increased AGE–RAGE-related inflammatory activity [75]. Circulating total sRAGE includes both proteolytically cleaved cRAGE and alternatively spliced esRAGE; therefore, elevated serum sRAGE may represent ligand sequestration, increased membrane-bound RAGE expression and shedding, or both [17,25]. These findings are consistent with the biological role of the AGE-RAGE axis in inflammatory signaling. Evidence concerning the relationship of sAGEs and sRAGE with inflammatory biomarkers in healthy adults remains limited. Consistent with the present findings, Jang et al. [80] reported a positive association between sAGEs and TNF-α in a large nondiabetic and nonobese population. However, clinical evidence remains inconsistent, as Dias et al. [81] found inverse correlations between sRAGE and TNF-α in patients with type 2 diabetes and diabetic nephropathy, whereas no significant association was observed among healthy controls. Positive associations of sRAGE with TNF-α and MCP-1 have been reported in patients with type 2 diabetes [82]. Liu et al. [83] reported inverse associations of sRAGE with TNF-α, IL-6, and IL-1β in patients with silicosis. Furthermore, Carrión-Barberà et al. [84] demonstrated associations of specific sAGEs and the CEL/sRAGE ratio with IL-6 in patients with systemic lupus erythematosus. Although the AGE–RAGE axis has been linked to inflammatory activity in several disease groups, evidence from healthy young adults remains limited. The positive association between sAGEs and inflammatory biomarkers may reflect increased endogenous glycation and AGE–RAGE-related activity under inflammatory conditions. However, circulating sAGE concentrations are also influenced by oxidative and metabolic processes, and this association cannot be attributed solely to RAGE activation [12]. Dietary AGE intake, estimated using the FFQ and 3D-FR, was not significantly associated with inflammatory or oxidative–antioxidant biomarkers. Dietary intervention studies in healthy or otherwise obese adults found that modifying dAGE intake did not significantly affect IL-6, TNF-α, CRP, or circulating sRAGE concentrations [67,85]. One possible explanation is that circulating AGE–RAGE concentrations may be influenced by endogenous glycation, metabolic processes, and inflammatory activity [12,28], although these mechanisms were not directly investigated in the present study. Furthermore, the lack of significant associations between dAGE intake or circulating AGE-RAGE biomarkers and antioxidant parameters does not provide clear evidence that dAGEs is directly related to antioxidant defence in this young adult sample.

### 4.3. sAGE–RAGE, Inflammation, and Attention/Working-Memory Performance in Separate Regression Models

Digit Span Backward requires both the maintenance and mental reordering of information and therefore places greater demands on working-memory manipulation than Digit Span Forward [54]. In contrast, Stroop Interference primarily reflects inhibitory control and the ability to resist cognitive interference [86].

In the separate models adjusted for age, sex, BMI, and depressive symptoms, after Benjamini–Hochberg FDR correction, higher circulating sAGE and TNF-α concentrations were significantly associated with lower Digit Span Backward scores, whereas no significant associations were observed for Digit Span Forward or Stroop Interference. This pattern may suggest a potential domain-specific association involving working-memory manipulation; however, direct comparisons between the cognitive outcomes were not performed. Because sAGE and TNF-α were examined in separate covariate-adjusted models, their associations with Digit Span Backward cannot be interpreted as mutually independent. Most previous studies have focused on older adults, diabetes, or individuals at increased risk of cognitive impairment. For example, greater AGE burden has been associated with poorer global cognition and domain-specific cognitive performance in older or clinically vulnerable populations [87,88,89]. Also, Deng et al. [38] reported associations between dAGE intake and cognitive performance across several domains.

Several interconnected biological processes may underlie these findings. Evidence from experimental and animal studies indicates that AGE–RAGE interaction can activate endothelial NF-κB signaling [90] and contribute to vascular dysfunction, while a high-AGE diet has been shown to induce brain AGE accumulation, mitochondrial dysfunction, and cognitive impairment in mice [91]. Experimental studies have also suggested that TNF-α may affect the neurovascular environment by disrupting cerebrovascular endothelial barrier integrity through IL-6 production and by stimulating brain pericytes to release IL-6, which can promote microglial activation [92,93]. These experimental findings provide potential biological context for the observed associations but do not demonstrate that these mechanisms occurred in the present study.

Dietary AGE intake, MDA, catalase, SOD, and GSH were not associated with any cognitive outcome in the present study. The absence of an association between dAGE intake and cognitive performance contrasts with the findings of Beeri et al. [39], who reported that higher dAGE intake was associated with faster cognitive decline in community-dwelling older adults. Lotan et al. [94] also found an inverse cross-sectional association between AGE intake and cognitive performance at baseline. However, global cognition improved in both the dAGEs reduction and standard care groups, preventing the observed improvement from being attributed specifically to reduced dAGE intake. Differences in age, duration of exposure, metabolic susceptibility, and study design may partly explain the discrepancy, as subtle dietary effects may be less readily detectable in healthy young adults than in aging populations or experimental models. Regarding redox biomarkers, Naseri et al. [95] reported no significant associations of circulating MDA or endogenous antioxidant markers with cognitive function in patients with multiple sclerosis. Similarly, Yang et al. [96] found no associations of plasma MDA, Mn-SOD, catalase, or GSH-Px activity with any cognitive domain among healthy unaffected adults. These findings are consistent with the absence of associations between MDA, SOD, catalase, and GSH and cognitive performance in the present study. In contrast, animal studies assessing redox status directly in brain or hippocampal tissue have reported that poorer working-memory performance is accompanied by increased MDA and reduced activities of antioxidant enzymes, including SOD and catalase [97,98]. Brain AGE accumulation and redox alterations may accompany cognitive impairment. In D-galactose-induced aging mice, Lian et al. [99] observed impaired learning and memory together with increased AGE and MDA levels and reduced catalase activity and total antioxidant capacity in the hippocampus and cortex. Notably, SOD and GPx activities did not differ significantly between the untreated model and control groups, suggesting that individual antioxidant markers may not respond uniformly despite concurrent AGE accumulation, lipid peroxidation, and cognitive impairment. These findings suggest that peripheral MDA and antioxidant markers may not be sufficiently sensitive to tissue-specific redox alterations associated with cognition. Therefore, the present findings do not exclude a contribution of dAGE intake or oxidative processes to cognitive function but indicate that such relationships were not detectable using the dietary and circulating biomarkers measured in this healthy young-adult sample.

### 4.4. PLS-SEM Structural Pathways Linking the AGE–RAGE–Inflammation Axis with Working Memory

The covariate-adjusted PLS-SEM model provided a complementary analytical perspective that was broadly consistent with the regression findings. Within the formative inflammation construct, IL-6 was the dominant indicator (outer weight = 0.808, *p* < 0.001; outer loading = 0.981). Although the outer weight of TNF-α was not statistically significant (outer weight = 0.260, *p* = 0.350), its substantial outer loading (0.796) indicated considerable absolute relevance, while collinearity was acceptable for both indicators (VIF = 1.781). In accordance with recommendations for evaluating formative measurement models, an indicator with a non-significant relative weight may be retained when its absolute contribution and theoretical relevance support its inclusion [58]. TNF-α was therefore retained because IL-6 and TNF-α have overlapping but biologically distinct inflammatory functions [100]. Nevertheless, the magnitude of the indicator weights indicates that the composite was predominantly driven by IL-6; accordingly, its association with cognitive performance should be interpreted as reflecting IL-6-weighted systemic inflammation rather than TNF-α-specific signaling. This interpretation is consistent with evidence that IL-6, but not TNF-α, was associated with default mode network connectivity in healthy adults [101]. Moreover, the formative construct included only IL-6 and TNF-α and therefore does not fully capture the complexity of systemic inflammatory activity [102].

Within the structural model, greater inflammation was associated with poorer Digit Span Backward performance. In the separate PLS-SEM models, inflammation was associated with poorer Digit Span Total performance, but not with Digit Span Forward or Stroop Interference. As Digit Span Total combines Forward and Backward scores, its association with inflammation may partly reflect the Backward component. These findings indicate that systemic inflammation may be particularly associated with the maintenance and mental reordering of information in working memory.

In the separate regression models, higher sAGEs and TNF-α concentrations were associated with poorer Digit Span Backward performance, whereas PLS-SEM identified an inverse association of the inflammation constructs with the same outcome. Working-memory performance may be influenced by inflammatory signaling within neural systems supporting the maintenance and manipulation of information. Circulating cytokines can communicate with the central nervous system through humoral, neural, and cellular pathways and may subsequently affect microglial activity, neurotransmitter metabolism, and synaptic plasticity [4]. Previous research examining IL-6 and TNF-α has also indicated that their relationships with cognition may vary according to the cognitive domain and population studied [103,104]. Lin et al. [103], for example, found that IL-6, but not TNF-α, was associated with poorer processing speed, whereas neither biomarker was associated with short-term memory in generally healthy adults. Longitudinally, Mac Giollabhui et al. [104] reported that chronic cumulative exposure to IL-6, TNF-α, and CRP, rather than inflammation assessed at a single time point, predicted worsening working-memory performance among adolescents and young adults. These findings support considering both biomarker-specific and cumulative inflammatory patterns when interpreting associations with cognition.

The direct path from sRAGE to Digit Span Backward was not statistically significant. In contrast, sRAGE showed an indirect inverse association with Digit Span Backward through inflammation, while serum AGEs were indirectly associated with greater inflammation through sRAGE. The indirect pathway from serum AGEs to Digit Span Backward through sRAGE alone was not supported. However, the serial indirect association from serum AGEs to Digit Span Backward through sRAGE and inflammation was supported. The results support a potential statistical link between circulating AGE–RAGE levels and poorer working-memory performance through inflammation within the specified structural model.

The absence of a direct sRAGE–cognition association and the presence of an indirect association through inflammation also emphasise that total sRAGE should not be interpreted solely as a protective decoy receptor. Although sRAGE can sequester circulating RAGE ligands, elevated concentrations may also reflect increased expression and proteolytic shedding of membrane-bound RAGE under conditions of greater ligand burden or inflammatory activity [24,25,28]. This may help contextualise why lower sRAGE has been interpreted as reduced decoy capacity in some chronic conditions, whereas higher sRAGE has been associated with inflammation, organ dysfunction, and adverse outcomes in others [25]. These indirect associations represent statistical decompositions of concurrent relationships under the prespecified model rather than temporal or causal mediation. Alternative biological sequences remain possible; inflammatory activity may, for example, promote membrane-RAGE shedding and increase circulating sRAGE rather than occurring exclusively downstream of sRAGE [25,28].

### 4.5. Strengths and Limitations

A major strength of this study is its integrated assessment of dietary AGE intake, circulating AGEs, sRAGE, inflammatory and oxidative–antioxidative biomarkers, and multiple cognitive outcomes in healthy young adults. Dietary AGE intake was estimated using both an FFQ and three-day food records, allowing habitual and short-term intake estimates to be considered separately. The analyses were also adjusted a priori for age, sex, BMI, and depressive symptoms, all of which may influence cognitive performance. In addition, combining conventional regression with PLS-SEM allowed both biomarker-specific associations and prespecified multivariable pathways to be examined.

Several limitations should be acknowledged as well. The cross-sectional design precludes determination of temporality and causality. The structural paths and indirect effects represent statistical decompositions of concurrent associations rather than biological mediation. The relatively small sample size may have limited statistical power, particularly for weak associations, multiple exposure–outcome comparisons, and complex structural pathways and indirect effects in the PLS-SEM analyses. Given the number of pathways examined, the PLS-SEM findings should also be interpreted as exploratory and require confirmation in larger independent samples. Although sex was included as a covariate in the adjusted analyses, the predominance of women may still limit the generalisability of the findings. Additionally, unmeasured lifestyle factors (e.g., recent caffeine intake, sleep duration, and physical activity), as well as dietary and socioeconomic factors, may have contributed to residual confounding. The negative Q^2^predict values in the PLS-SEM analysis indicated limited out-of-sample predictive relevance. As prediction was not a primary objective of the study, these findings should be interpreted within the context of the proposed structural model. Moreover, dAGEs estimation is subject to measurement error arising from self-reported intake and the incomplete coverage of AGE databases. The database was not independently validated for locally consumed foods and recipes in this study, and differences in food composition and cooking conditions may have affected the accuracy of dAGEs estimation. sAGE assays may quantify a heterogeneous mixture of compounds and may not distinguish specific AGEs such as carboxymethyl-lysine, methylglyoxal-derived hydroimidazolone, or pentosidine. Similarly, total sRAGE measurement did not distinguish cRAGE from esRAGE, limiting mechanistic interpretation. Finally, cognition was assessed using Digit Span and Stroop tests rather than a comprehensive neuropsychological battery. The findings may therefore not be generalisable to broader domains such as episodic memory, learning, language or global cognition.

## 5. Conclusions

In healthy young adults, dietary AGE intake estimated using the FFQ and the three-day food record was not associated with circulating AGE concentrations. Higher serum AGE and TNF-α concentrations were associated with poorer Digit Span Backward performance after FDR correction, while no significant associations were observed for Digit Span Forward or Stroop Interference. Consistently, PLS-SEM identified an inverse association between systemic inflammation and Digit Span Backward performance and indicated indirect associations linking sRAGE to working memory through inflammation. These findings suggest associations of circulating AGE–RAGE-related biomarkers and inflammatory activity with the maintenance and mental reordering of information in working memory among healthy young adults, while no corresponding associations were detected for estimated dietary AGE intake. Given the cross-sectional design, these findings should be interpreted as associations and not as evidence of a causal AGE–RAGE–inflammation–cognition pathway. Longitudinal studies with larger and more representative samples are required to confirm these findings and clarify their temporal and biological significance.

## Figures and Tables

**Table 1:** General Characteristics of Participants (N = 90).

Category	Variable	Value ^a^
Socio-demographic and anthropometric characteristics	Sex (female)	66 (73.3%)
	Age (years)	22.2 ± 1.3
	Income Level (Income = Expense)	60.0 (66.7)
	BMI (kg/m^2^)	22.2 ± 3.2
Dietary AGE Intake	FFQ-dAGEs (kU/day)	14,067.7 ± 6516.8
	3D-FR-dAGES (kU/day)	10,168.5 ± 5409.7
Biochemical Markers	sAGEs (ng/mL)	308.9 ± 189.6
	sRAGE (pg/mL)	1140.9 ± 787.9
	TNF-α (ng/L)	157.1 [123.5]
	IL-6 (ng/L)	16.0 [8.2]
	SOD (U/mL)	96.7 ± 8.7
	GSH (mM)	0.6 ± 0.1
	MDA (µM)	5.2 ± 1.5
	Catalase (U/mL)	2751.0 ± 761.5
Cognitive Outcomes	Digit Span Forward	7.0 ± 1.6
	Digit Span Backward	6.7 ± 2.4
	Digit Span Total	13.8 ± 3.6
	Stroop Interference (s)	18.1 ± 3.9
	BDI-II score	11 ± 7.4

BDI-II, Beck Depression Inventory-II; BMI, body mass index; FFQ, food frequency questionnaire; GSH, glutathione; IL-6, interleukin-6; MDA, malondialdehyde; sAGEs, serum advanced glycation end products; sRAGE, soluble receptor for advanced glycation end products; SOD, superoxide dismutase; TNF-α, tumour necrosis factor-alpha; 3D-FR-dAGEs, dietary advanced glycation end products estimated from 3-day food records; FFQ-dAGEs, dietary advanced glycation end products estimated from the food frequency questionnaire. ^a^ Continuous variables are presented as mean ± standard deviation (SD) or median [interquartile range (IQR)], as appropriate; categorical variables are presented as *n* (%).

## Data Availability

The data presented in this study are available on request from the corresponding author due to ethical and participant privacy considerations.

## Supplementary Materials

The following supporting information can be downloaded at https://www.mdpi.com/article/10.3390/nu18203442/s1. Table S1. Assessment of the formative measurement model for inflammation. Table S2. Complete adjusted structural model for Digit Span Total. Table S3. Complete adjusted structural model for Digit Span Forward. Table S4. Complete adjusted structural model for Stroop interference. Table S5. Summary of covariate-adjusted PLS-SEM associations across cognitive outcomes. Table S6. Out-of-sample predictive assessment using PLSpredict and CVPAT. Table S7. Standardized root mean square residual across cognitive models. Table S8. Complete adjusted structural model for Digit Span Backward.
