# Supplementary material for: Dietary and Circulating Advanced Glycation End Products in Relation to Inflammation and Cognitive Function in Healthy Young Adults
Source: Nutrients. 2026 Oct 20;18(20):3442. doi: 10.3390/nu18203442 (PMC13708714; doi:10.3390/nu18203442)
Supplement: Supplementary file 1 [file nutrients-18-03442-s001.zip › nutrients-4613871-supplementary.pdf]

PLS-SEM Manuscript Tables

Supplementary Table S1. Assessment of the formative measurement model for inflammation.

Indicator	Outer weight (w)	SE	t	p	Outer loading	VIF	Decision
IL-6	0.808	0.229	3.537	<0.001	0.981	1.781	Retained
TNF-α	0.260	0.279	0.935	0.350	0.796	1.781	Retained[1]

Note. VIF, variance inflation factor. [1]TNF-α was retained despite its non-significant outer weight because of its theoretical relevance and satisfactory absolute outer loading.

Supplementary Table S2. Complete adjusted structural model for Digit Span Total.

Section	Path	β	SE	t	p	95% bootstrap CI	f^2
Hypothesised paths	FFQ-dAGEs -> Serum AGEs	0.073	0.138	0.528	0.597	-0.208 to 0.332	0.005
	3D-FR-dAGEs -> Serum AGEs	-0.034	0.113	0.299	0.765	-0.250 to 0.193	0.001
	Inflammation -> Digit Span Total	-0.308	0.145	2.125	0.034	-0.596 to -0.025	0.065
	Serum AGEs -> sRAGE	0.505	0.106	4.754	<0.001	0.263 to 0.674	0.342
	sRAGE -> Inflammation	0.601	0.099	6.052	<0.001	0.385 to 0.759	0.640
	sRAGE -> Digit Span Total	0.089	0.134	0.665	0.506	-0.170 to 0.356	0.006
Covariate paths	BMI -> Inflammation	-0.107	0.104	1.023	0.306	-0.291 to 0.119	0.015
	BMI -> Digit Span Total	-0.071	0.117	0.609	0.542	-0.295 to 0.164	0.004
	Sex -> Inflammation	-0.066	0.302	0.218	0.827	-0.597 to 0.596	0.001
	Sex -> Digit Span Total	-0.855	0.247	3.456	<0.001	-1.353 to -0.361	0.132
	Depressive symptom score -> Inflammation	-0.147	0.080	1.836	0.066	-0.288 to 0.026	0.038
	Depressive symptom score -> Digit Span Total	-0.084	0.097	0.867	0.386	-0.270 to 0.115	0.008
	Age -> Inflammation	0.346	0.106	3.280	0.001	0.149 to 0.555	0.205
	Age -> Digit Span Total	0.076	0.098	0.775	0.438	-0.123 to 0.263	0.006

Note. Note. Sex was coded as 0 = female and 1 = male.β values are standardized path coefficients; SE, bootstrap standard error; t, bootstrap t statistic; CI, confidence interval; f^2, local effect size; BMI, body mass index; sAGEs, serum advanced glycation end products; sRAGE, soluble receptor for advanced glycation end products; FFQ-dAGEs, dietary AGEs estimated using a food-frequency questionnaire; 3D-FR-dAGEs, dietary AGEs estimated using a three-day food record. The model was adjusted for age, sex, BMI, and depressive symptom score. Confidence intervals are percentile bootstrap confidence intervals based on 10,000 bootstrap resamples.

Supplementary Table S3. Complete adjusted structural model for Digit Span Forward.

Section	Path	β	SE	t	p	95% bootstrap CI	f²
Hypothesised paths	FFQ-dAGEs -> Serum AGEs	0.073	0.138	0.528	0.597	-0.208 to 0.332	0.005
	3D-FR-dAGEs -> Serum AGEs	-0.034	0.113	0.299	0.765	-0.250 to 0.193	0.001
	Inflammation -> Digit Span Forward	-0.099	0.162	0.615	0.539	-0.404 to 0.235	0.006
	Serum AGEs -> sRAGE	0.505	0.106	4.754	<0.001	0.263 to 0.674	0.342
	sRAGE -> Digit Span Forward	0.019	0.136	0.136	0.892	-0.244 to 0.291	0.000
	sRAGE -> Inflammation	0.601	0.104	5.765	<0.001	0.367 to 0.759	0.639
Covariate paths	BMI -> Digit Span Forward	-0.145	0.114	1.279	0.201	-0.359 to 0.090	0.017
	BMI -> Inflammation	-0.103	0.107	0.968	0.333	-0.289 to 0.129	0.014
	Sex -> Digit Span Forward	-0.712	0.236	3.018	0.003	-1.161 to -0.225	0.082
	Sex -> Inflammation	-0.052	0.322	0.162	0.871	-0.632 to 0.639	0.001
	Depressive symptom score -> Digit Span Forward	-0.011	0.099	0.107	0.915	-0.198 to 0.187	0.000
	Depressive symptom score -> Inflammation	-0.150	0.080	1.872	0.061	-0.296 to 0.017	0.040
	Age -> Digit Span Forward	-0.088	0.115	0.771	0.441	-0.321 to 0.128	0.007
	Age -> Inflammation	0.345	0.105	3.282	0.001	0.147 to 0.551	0.203

Note. Sex was coded as 0 = female and 1 = male.β values are standardized path coefficients; SE, bootstrap standard error; t, bootstrap t statistic; CI, confidence interval; f², local effect size; BMI, body mass index; sAGEs, serum advanced glycation end products; sRAGE, soluble receptor for advanced glycation end products; FFQ-dAGEs, dietary AGEs estimated using a food-frequency questionnaire; 3D-FR-dAGEs, dietary AGEs estimated using a three-day food record. The model was adjusted for age, sex, BMI, and depressive symptom score. Confidence intervals are percentile bootstrap confidence intervals based on 10,000 bootstrap resamples.

Supplementary Table S4. Complete adjusted structural model for Stroop interference.

Section	Path	β	SE	t	p	95% bootstrap CI	f²
Hypothesised paths	FFQ-dAGEs -> Serum AGEs	0.073	0.138	0.528	0.597	-0.208 to 0.332	0.005
	3D-FR-dAGEs -> Serum AGEs	-0.034	0.113	0.299	0.765	-0.250 to 0.193	0.001
	Inflammation -> Stroop interference	-0.051	0.169	0.302	0.762	-0.381 to 0.284	0.001
	Serum AGEs -> sRAGE	0.505	0.106	4.754	<0.001	0.263 to 0.674	0.342
	sRAGE -> Stroop interference	0.034	0.134	0.256	0.798	-0.231 to 0.293	0.001
	sRAGE -> Inflammation	0.601	0.105	5.750	<0.001	0.364 to 0.758	0.640
Covariate paths	BMI -> Stroop interference	-0.020	0.112	0.176	0.860	-0.240 to 0.204	0.000
	BMI -> Inflammation	-0.108	0.107	1.014	0.311	-0.288 to 0.134	0.015
	Sex -> Stroop interference	0.086	0.318	0.272	0.786	-0.521 to 0.727	0.001
	Sex -> Inflammation	-0.072	0.309	0.232	0.816	-0.625 to 0.608	0.001
	Depressive symptom score -> Stroop interference	0.049	0.118	0.410	0.682	-0.181 to 0.277	0.002
	Depressive symptom score -> Inflammation	-0.145	0.083	1.748	0.081	-0.301 to 0.021	0.037
	Age -> Stroop interference	0.020	0.133	0.148	0.882	-0.237 to 0.284	0.000
	Age -> Inflammation	0.347	0.107	3.228	0.001	0.140 to 0.553	0.206

Note. Sex was coded as 0 = female and 1 = male.β values are standardized path coefficients; SE, bootstrap standard error; t, bootstrap t statistic; CI, confidence interval; f², local effect size; BMI, body mass index; sAGEs, serum advanced glycation end products; sRAGE, soluble receptor for advanced glycation end products; FFQ-dAGEs, dietary AGEs estimated using a food-frequency questionnaire; 3D-FR-dAGEs, dietary AGEs estimated using a three-day food record. The model was adjusted for age, sex, BMI, and depressive symptom score. Confidence intervals are percentile bootstrap confidence intervals based on 10,000 bootstrap resamples.

Supplementary Table S5. Summary of covariate-adjusted PLS-SEM associations across cognitive outcomes.

Outcome	Inflammation → outcome β	SE	p	95% bootstrap CI	Outcome R^2
Backward	-0.398	0.152	0.009	-0.697 to -0.110	0.234
Total	-0.308	0.145	0.034	-0.596 to -0.025	0.184
Forward	-0.099	0.162	0.539	-0.404 to 0.235	0.092
Stroop interference	-0.051	0.169	0.762	-0.381 to 0.284	0.007

Note. All models were adjusted for age, sex, BMI, and depressive symptom score.

Supplementary Table S6. Out-of-sample predictive assessment using PLSpredict and CVPAT.

Outcome	Q²predict	PLS-RMSE	LM-RMSE	PLS-MAE	LM-MAE	CVPAT PLS–LM p
Backward	-0.261	2.690	2.433	2.051	1.977	0.057
Total	-0.330	4.092	3.621	3.227	2.953	0.030
Forward	-0.264	1.846	1.701	1.477	1.387	0.097
Stroop interference	-0.100	4.139	4.178	3.413	3.373	0.718

Note. PLSpredict and CVPAT used 10-fold cross-validation with 10 repetitions and a fixed seed. SE, standard error; CI, confidence interval; RMSE, root mean square error; MAE, mean absolute error; LM, linear model; CVPAT, cross-validated predictive ability test.

Supplementary Table S7. Standardized root mean square residual across cognitive models.

Model	Saturated-model SRMR	Estimated-model SRMR
Backward	0.036	0.106
Total	0.038	0.109
Forward	0.040	0.101
Stroop interference	0.039	0.083

Note. SRMR, standardized root mean square residual. Saturated- and estimated-model SRMR values are shown separately.

Supplementary Table S8. Complete adjusted structural model for Digit Span Backward.

Section	Path	β	SE	t	p	95% bootstrap CI	f²
Hypothesised paths	FFQ-dAGEs → sAGEs	0.073	0.138	0.528	0.597	−0.208 to 0.332	0.005
	3D-FR-dAGEs → sAGEs	−0.034	0.113	0.299	0.765	−0.250 to 0.193	0.001
	sAGEs → sRAGE	0.505	0.106	4.754	<0.001	0.263 to 0.674	0.342
	sRAGE → Inflammation	0.601	0.099	6.070	<0.001	0.383 to 0.758	0.638
	Inflammation → Digit Span Backward	−0.398	0.152	2.617	0.009	−0.697 to −0.110	0.115
	sRAGE → Digit Span Backward	0.125	0.142	0.881	0.379	−0.154 to 0.404	0.012
Covariate paths	Age → Inflammation	0.350	0.107	3.280	0.001	0.149 to 0.557	0.209
	Age → Digit Span Backward	0.178	0.108	1.646	0.100	−0.036 to 0.390	0.033
	Sex → Inflammation	−0.098	0.295	0.333	0.739	−0.619 to 0.543	0.003
	Sex → Digit Span Backward	−0.792	0.256	3.097	0.002	−1.325 to −0.298	0.121
	BMI → Inflammation	−0.114	0.104	1.097	0.273	−0.300 to 0.109	0.017
	BMI → Digit Span Backward	−0.010	0.119	0.086	0.932	−0.239 to 0.232	0.000
	Depressive symptom score → Inflammation	−0.139	0.081	1.709	0.088	−0.281 to 0.039	0.034
	Depressive symptom score → Digit Span Backward	−0.116	0.088	1.316	0.188	−0.281 to 0.065	0.017

Note. Sex was coded as 0 = female and 1 = male.β values are standardized path coefficients; SE, bootstrap standard error; t, bootstrap t statistic; CI, confidence interval; f², local effect size; BMI, body mass index; sAGEs, serum advanced glycation end products; sRAGE, soluble receptor for advanced glycation end products; FFQ-dAGEs, dietary AGEs estimated using a food-frequency questionnaire; 3D-FR-dAGEs, dietary AGEs estimated using a three-day food record. The model was adjusted for age, sex, BMI, and depressive symptom score. Confidence intervals are percentile bootstrap confidence intervals based on 10,000 bootstrap resamples.
